# Heteroarylketones inhibit astroglial interleukin-6 expression via a STAT3/NF-κB signaling pathway

**DOI:** 10.1186/1742-2094-8-86

**Published:** 2011-07-29

**Authors:** Ingo Schulz, Claudia Engel, André J Niestroj, Ulrike Zeitschel, Katja Menge, Astrid Kehlen, Antje Meyer, Steffen Roßner, Hans-Ulrich Demuth

**Affiliations:** 1 Probiodrug AG, Weinbergweg 22, Halle/Saale, 06120, Germany; 2 Department of Cellular and Molecular Mechanisms of Neurodegeneration, Paul Flechsig Institute for Brain Research, University of Leipzig, Jahnallee 59, Leipzig, 04109, Germany

**Keywords:** astrocytes, LPS mouse model, IL-6 expression, anti-inflammatory, STAT3 phosphorylation, p65 co-immunoprecipitation

## Abstract

**Background:**

Elevated brain levels of the pleiotropic cytokine interleukin-6, which is mainly secreted from activated local astrocytes, contribute to pathological events including neuroinflammation and neurodegeneration. Thus, inhibition of pathological IL-6 expression provides a rationale strategy for targeting the onset or further progression of neurological disorders including Alzheimer's disease, multiple sclerosis, Parkinson's disease and traumatic brain injury. The purpose of this study was to identify and to characterize new potent inhibitors of astrocytic IL-6 expression for further therapeutic development of novel anti-inflammatory and neuroprotective drugs.

**Methods:**

Oncostatin M (OSM)-treated human glioma U343 cells were used as model for induction of astrocytic IL-6 expression. This model was characterized by immunoblotting, siRNA technique, ELISA and qRT-PCR and used to screen low molecular weight compound libraries for IL-6-lowering effects. To validate bioactive compounds identified from library screens, bacterial lipopolysaccharide was used to induce IL-6 expression in cultivated primary astrocytes and in mice *in vivo*. To dissect underlying molecular mechanisms, protein extracts from OSM-treated U343 cells were analyzed by phospho-specific immunoblotting and immunocytochemistry as well as by co-immunoprecipitation.

**Results:**

OSM-treatment (100 ng/ml; 24 h) led to 30-fold increase of IL-6 secretion from U343 cells. The temporal profile of IL-6 mRNA induction displayed a biphasic induction pattern with peak synthesis at 1 h (6.5-fold) and 16 h (5.5-fold) post stimulation. IL-6 protein release did not show that biphasic pattern and was detected as early as 3 h post stimulation reaching a maximum at 24 h. The screen of compound libraries identified a set of heteroarylketones (HAKs) as potent inhibitors of IL-6 secretion. HAK compounds affected the second peak in IL-6 mRNA synthesis, whereas the first peak was insensitive to HAK treatment. HAK compounds also suppressed lipopolysaccharide-induced IL-6 expression in primary murine astrocytes as well as in brain and plasma samples from lipopolysaccharide-treated mice. Finally, HAK compounds were demonstrated to specifically suppress the OSM-induced phosphorylation of STAT3 at serine 727 and the physical interaction of pSTAT3^S727 ^with p65.

**Conclusion:**

Heteroarylketone compounds are potent inhibitors of IL-6 expression *in vitro *and *in vivo *and may represent a new class of potent anti-inflammatory and neuroprotective drugs.

## Background

Interleukin-6 (IL-6) is a pleiotropic cytokine involved in several brain diseases as a detrimental factor playing a causal or exacerbating role in neuroinflammation and neurodegeneration [1-7]. Elevated levels of IL-6 are typical for brains from animal models or humans suffering from multiple sclerosis, Alzheimer's disease, Parkinson's disease, lethal sepsis, meningitis and stroke [8-12]. Additionally, long-term exposure of neurons or astrocytes to IL-6 as well as over-activation of IL-6 signaling by IL-6/sIL-6R fusion protein lead to a robust induction of neuroinflammatory response and to neuronal death [6,13-16]. Therefore, suppression of IL-6 signaling or of IL-6 expression itself is believed to represent a powerful strategy for the treatment or prevention of neuroinflammation and subsequent neurodegeneration. This is supported by diminished neuroinflammation induced by spinal cord injury after infusion of a monoclonal antibody against IL-6 receptor [17]. Furthermore, the potency of drugs to inhibit IL-6 expression *in vitro *and *in vivo *correlates with their anti-neuroinflammatory and neuroprotective properties [18-22].

Astrocytes, the main glial cell type of the brain, respond in general to multiple kinds of acute and chronic brain insults with a reaction known as astrogliosis [23]. This reactive astrogliosis involves morphological, structural and biochemical features including thickened cellular processes, increased expression of glial fibrillary protein and the induction of pro-inflammatory cytokines including IL-6 [24,25]. Different types of signaling molecules are able to trigger the astrocytic IL-6 mRNA expression via distinct intracellular signaling pathways [26]. For example, lipopolysaccharide (LPS) activates the IL-1 receptor-associated kinase (IRAK)-dependent pathway including IκB kinase and nuclear factor κB (NF-κB) [27]. Another potent group of IL-6 inducers are cytokines such as tumor necrosis factor α, interleukin-1β, oncostatin M (OSM) and leukaemia inhibitory factor (LIF) [28-30]. Interestingly, OSM and LIF belong together with IL-6 to the same cytokine family. These IL-6-type cytokines are characterized by using of glycoprotein gp130 to induce gene expression via JAK/STAT (Janus kinase/signal transducer and activator of transcription) and MAPK (mitogen-activated protein kinase) cascades in a NF-κB-dependent manner [31,32]. Thus, blocking of such pathological IL-6-driven gene expression by low molecular weight inhibitors provides a possible strategy for targeting the onset or further propagation of astrogliosis and, subsequently, secondary neuronal cell death.

In the present study, the time- and dose-dependent stimulation of IL-6 expression by OSM was characterized in human U343 glioma cells. Subsequently, our compound libraries were screened for inhibitory effects on OSM-induced IL-6 expression. We identified bioactive compounds belonging to the chemical class of heteroarylketones (HAK). These HAK compounds were able to suppress the LPS-induced IL-6 expression in primary mouse and rat astrocytes as well as in an acute septic shock mouse model *in vivo*. Finally, the underlying molecular mechanism of HAK compounds interfering with key signaling molecules of OSM-induced signal transduction cascade was analyzed. We demonstrate a selective suppression by HAK compounds of the OSM-mediated phosphorylation of STAT3 at serine 727, which affects STAT3 binding to the NF-κB subunit p65.

## Methods

### Primary cultures of murine astrocytes

According to Löffler [33], astrocyte-rich primary cell cultures were started with brains of newborn mice and rats and were maintained in Dulbecco's modified Eagle's medium (DMEM) for 33 days at 37°C in a humified atmosphere with 95% air/5% CO_2_.

### Cell culture

The human glioma cell line U343 [34] was maintained in DMEM containing 10% fetal bovine serum and 60 μg/ml gentamycin (Invitrogen, Darmstadt, Germany) at 37°C in a 10% CO_2 _atmosphere.

### Lipopolysaccharide (LPS)-induced acute septic shock model

A total number of 20 C57/B6 mice at the age of 5 months with an initial body weight of 22 g to 30 g was analyzed in this study. Mice were randomly assigned to 2 groups of control mice (injection of saline or LPS; n = 6 each) and 1 group of LPS-treated mice co-injected with compound HAK-2 (n = 8). Animals were housed at the Experimental Animal Core Facility of the University of Leipzig. The study was approved by the Regierungspräsidium Leipzig, License # TVV 28/07 on November 14, 2007. To induce septic shock and acute inflammation, 14 mice were injected intraperitoneally (i.p.) with 1 mg LPS (Serotyp 055:B5)/kg body weight. Saline injection i.p. was used as control treatment (n = 6). Compound HAK-2 was co-injected i.p. to 8 LPS-treated mice at a concentration of 10 mg/kg body weight. Two hours post treatment mice were sacrificed by CO_2 _inhalation and tissue samples from hippocampus and cortex as well as plasma were prepared as described later.

Mouse brain samples for qRT-PCR were prepared from 6 - 8 animals per group. After removal of the brain, the tissue samples were flushed shortly with ice cold saline and placed briefly on filter paper. Cortex and hippocampus were prepared, weighted and snap frozen in liquid nitrogen. Samples were stored at -80°C until RNA isolation. Frozen tissue samples were homogenized in RNA lysis buffer (Macherey and Nagel, Düren, Germany) using Precellys ceramic beads (diameter of 1.4 mm; Peqlab, Erlangen, Germany).

At the end of the experiment blood samples were collected in ice-cooled tubes and centrifuged within the next 20 min (10 min, 1500 × g, 4°C). Plasma samples were aliquoted into fractions of 50 μl, shock frozen and stored at -80°C until analysis.

### Quantitative real-time PCR

Total RNA of cultured cells and homogenized mouse brain tissue samples was isolated using RNA isolation kit "NucleoSpin RNA II" from Macherey and Nagel. RNA quantity was measured by spectrophotometrical quantification (NanoDrop 2000, Peqlab, Erlangen, Germany). Total RNA (0.5 μg) was transcribed to cDNA using Superscript III and oligo-dT primer (Invitrogen, Darmstadt, Germany). Quantitative real-time PCR was performed with QuantiFast SYBR Green PCR Master mix (Qiagen, Hilden, Germany) using Rotorgene 3000 system (Corbett, Sydney, Australia). Gene expression was normalized to the expression of three reference genes glyceraldehyde 3-phosphate dehydrogenase (GAPDH), glucose-6-phosphate dehydrogenase (G6PDH) and hypoxanthine-guanine phosphoribosyltransferase (HPRT). Primers for PREP (5'-TGAGCAGTGTCCCATCAGAG-3'; 5'- CATCTTCGCTGAACGCATAA-3') and IL-6 (5'-AAAGGACGTCACATTGCA-3'; 5'-GCCTCAGACATCTCCAGTCC-3') were designed using the Primer3 Software (http://frodo.wi.mit.edu/). Data were analyzed by Rotorgene 3000 Software version 6.0 by comparative quantitation. The appropriate size of PCR products was confirmed by gel-electrophoresis stained with ethidium bromide.

### ELISA

Measurements of IL-6 in conditioned medium and murine plasma samples were done by means of human- or murine-specific IL-6 Cytoset kits (Invitrogen, Darmstadt, Germany) according to manufacturer's protocols.

### Preparation of whole-cell extracts for western blotting and immunoprecipitation

Human U343 cells were lysed with cell lysis buffer (Invitrogen, Darmstadt, Germany) supplemented with 0.2 mg/ml sodium orthovanadate (Sigma, Taufkirchen, Germany), protease inhibitor mix complete mini (Roche, Mannheim, Germany) and 1 mM AEBSF (Sigma, Taufkirchen, Germany) for 30 min on ice. Lysates were centrifuged for 15 min at 15,000 × g and 4°C. Protein content in supernatants was quantified by Bradford assay (Sigma, Taufkirchen, Germany) according to the manufacturer's protocol and stored at -20°C.

### Western blotting

Western blotting was performed with 30 μg protein of whole-cell extracts, mixed with 4 x SDS sample loading buffer (Invitrogen, Darmstadt, Germany) and denatured for 10 min at 85°C. Cell extracts separated by 4 - 12% Novex Bis-Tris Mini Gel system (Invitrogen, Darmstadt, Germany) were transferred to Roti-NC nitrocellulose membranes (Roth, Karlsruhe, Germany). Membranes were probed with primary antibodies against STAT3 (#9132) and P-STAT3^S727 ^(#9134) from Cell Signaling (Frankfurt/M., Germany) as well as with anti-αtubulin (Sigma, Taufkirchen, Germany) to confirm equal loading and blotting of protein samples. Proteins were visualized using HRP-conjugated secondary antibodies (1:4000, cell signaling, Frankfurt/M., Germany) and the SuperSignal West Pico system (Thermo Fisher Scientific, Karlsruhe, Germany).

### Small interfering RNA (siRNA)

Human U343 cells (0.25 × 10^6^) were seeded in 24-well plates (Greiner, Frickenhausen, Germany) and transfected immediately with 2.5 μl A. dest. (mock), 100 μM non-target control (NTC) or PREP-specific siRNA ON-TARGETplus SMARTpool (#L-006006-00, Dharmacon, Schwerte, Germany) using DharmaFECT 1 siRNA transfection reagent (Dharmacon, Schwerte, Germany) according to the manufacturer's protocol. After 48 h adherent cells were transfected a second time under identical conditions for further 24 h and subsequently stimulated with OSM (100 ng/ml) for additional 6 h. For IL-6 specific ELISA 5 - 40 μl of conditioned media were utilized. To analyze IL-6 mRNA expression by qRT-PCR, total RNA was isolated and reversely transcribed as described above.

### Immunocytochemistry

Human U343 cells (1 × 10^5 ^cells/well) were grown on cover slips in 24-well plates (Greiner, Frickenhausen, Germany) for 24 h. After the time of treatment indicated cells were fixed in ice-cold methanol for 10 min on ice, and then incubated with rabbit anti-phospho-STAT3 antibody (#9134, Cell Signaling, Frankfurt/M., Germany) overnight at 8°C. Subsequently, cells were incubated with goat anti-rabbit IgG Cy2-conjugated secondary antibody (Dianova, Hamburg, Germany) at room temperature for 45 min. Finally, cover slips were mounted on microscope slides and approximately 250 cells/sample were evaluated densitometrically by fluorescence microscopy (Zeiss, Jena, Germany) and MetaMorph Image Analysis Software (Universal Imaging Corporation, USA).

### Immunoprecipitation

Cell lysates from 4 × 10^6 ^U343 cells/sample were obtained as described above. From each sample 200 μg of total protein were immunoprecipitated with 2 μg rabbit anti-p65 antibody (Santa Cruz Biotechnology; Heidelberg, Germany) or A. dest. (control) overnight at 4°C. Immunoprecipitated samples were incubated with 20 μl Dynabeads Protein G (Thermo Fisher Scientific, Karlsruhe, Germany) for 1 h at 4°C. Beads were washed 3 times with 1 ml PBS. Preparation of samples for SDS-PAGE analysis was done by means of dilution with SDS-PAGE sample buffer (Invitrogen, Darmstadt, Germany), followed by denaturation at 90°C for 10 min. SDS-PAGE analysis were performed as described before.

### PREP enzymatic activity assay

The activity of human recombinant prolyl endopeptidase (PREP) was determined photometrically using the substrate Z-Gly-Pro-pNA (Bachem, Weil am Rhein, Germany) usually at a concentration of 150 μM. As assay buffer 50 mM HEPES buffer pH 7.6, containing 200 mM NaCl, 1 mM EDTA, and 1 mM dithiothreitol was used. Release of pNA was monitored continuously at 405 nm for 15 min at 30°C in a 96-well plate reader (Spectrafluor, Tecan, Crailsheim, Germany). PREP activity was calculated from the slope of the time product curve with the help of a pNA standard [35]. For determination of Ki values three different substrate concentrations (62.5 μM; 125 μM and 250 μM) and seven different inhibitor concentrations were analyzed. Substrate concentrations were selected to be at the Km value of Z-Gly-Pro-pNA (125 μM) as well as half and twice the Km. Data were fitted by non-linear regression to the competitive inhibitor equation (Graphit 5.0, Erithacus software).

### Statistical analyses

Values are expressed as mean ± SD. Standard unpaired t test was used for analyses of statistically significance. Differences between treatments were considered significant when p < 0.05.

## Results

### Oncostatin M-mediated release of IL-6 in human U343 glioma cells

Beside myocytes and adipocytes, glial cells are representing the most prominent source of the cytokine IL-6 in mammals and play an important role in neuroinflammatory processes [23,36]. Therefore, the human glioma cell line U343 was selected for screening of IL-6-lowering effects of our in-house compound libraries. Preceding experiments with a set of stimuli known from literature to induce IL-6 expression in astroytes [30,37], identified Oncostatin M (OSM) as a robust inductor of IL-6 protein release in our experimental setup (data not shown).

**Figure 1:**
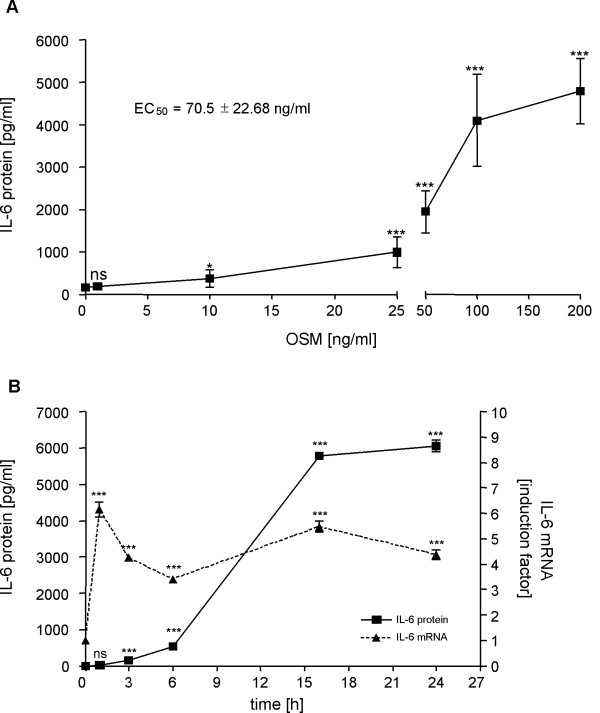
**OSM induces dose- and time-dependent IL-6 expression in human U343 glioma cells**. OSM treatment leads to dose-dependent IL-6 release (A). U343 cells were stimulated with different concentrations of OSM as indicated. After 24 hours the conditioned medium was analyzed by IL-6-specific ELISA. The time-dependent profile of OSM treatment shows a significant increase in IL-6 mRNA expression and protein secretion (B). U343 cells were stimulated with 100 ng/ml OSM and harvested at time points indicated to extract total RNA or whole cell lysate. IL-6 mRNA expression was analyzed by qRT-PCR (dotted line, black triangles) and IL-6 protein release by ELISA (solid line, black squares). All data points, presented as mean ± SD, are from experiments carried out in quadruplicate. ns, not significant; *p < 0.05; ***p < 0.001.

The dose- and time-dependent stimulation of IL-6 expression by OSM in U343 cells is characterized in figure 1. To analyze dose dependence of IL-6 release, U343 cells were treated for 24 hours with various concentrations of OSM followed by measurement of IL-6 protein concentrations in the conditioned medium by a specific ELISA. OSM induced the release of IL-6 in a dose-dependent manner with an EC_50 _of 70.5 ± 22.68 ng/ml (Figure 1A). Therefore, all further investigations were performed with 100 ng/ml OSM. The highest dose of 200 ng/ml OSM led to 30-fold increase of IL-6 accumulation in the conditioned media in comparison to vehicle-treated cells.

To analyze the time-course of OSM-induced IL-6 expression, U343 cells were incubated with OSM for different periods of time as indicated in figure 1B. Amounts of IL-6 mRNA and protein were subsequently quantified by qRT-PCR and by ELISA, respectively. Time course studies revealed that IL-6 mRNA displays a biphasic induction pattern with peak synthesis at 1 h (6.5-fold) and 16 h (5.5-fold) post stimulation. Significant induction of IL-6 protein was detected in the conditioned medium as early as 3 h post stimulation and reaching a maximum at 24 h. In contrast to the mRNA expression profile, IL-6 protein release did not show a biphasic pattern.

### Identification of compounds reducing OSM-induced IL-6 release in human U343 glioma cells

**Figure 2:**
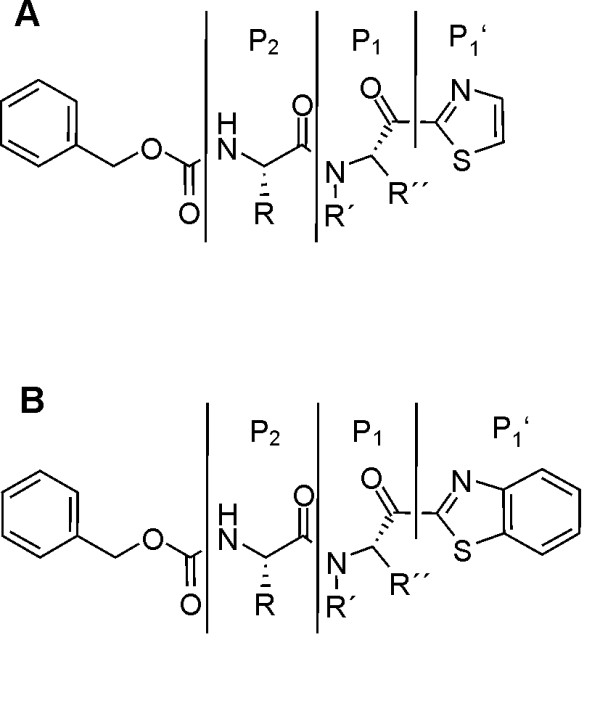
**General structures of IL-6 lowering HAK compounds**. All bioactive compounds are structurally related and belong to the class of HAKs containing either thiazole (A; -Th) or benzothiazole (B; -BTh) as reactive moiety at P_1_'.

Using the characterized cell culture model of OSM-induced IL-6 expression, our in-house compound libraries were screened for potent IL-6 expression inhibitors. Human U343 glioma cells were treated with 100 ng/ml OSM for 24 h. The analysis by IL-6 ELISA identified a set of structurally related compounds as potent inhibitors of IL-6 secretion (Table 1). Interestingly, all bioactive compounds identified belong to the class of heteroarylketones (HAK) and differ from each other at residues R to R'' and P_1_, P_1_' and P_2_, respectively (Figure 2). HAKs with proline in position P_1 _are known from the literature as inhibitors of prolyl endopeptidase (PREP), a proline-specific serine protease [38]. Determination of the Ki values proved that compounds with proline in position P_1 _are highly potent inhibitors of PREP. The remaining compounds with a substituted moiety in that region showed very poor PREP inhibition (Table 1).

**Table 1:** Effects of HAK compounds on PREP inhibition and on OSM-stimulated IL-6 secretion from U343 cells

Compound	Z-P_2_-P_1_-P_1_'	Remaining IL-6 expression [% of control]	PREP assay (Ki [M]
HAK-1	Z-Phe-Pro-BTh	**15.9 ± 1.87**	**4.6 × 10^-9^**

HAK-2	Z-Ala-Pro-BTh	**18.7 ± 3.87**	**3.8 × 10^-11^**

HAK-3	Z-Phe-Leu-BTh	**22.1 ±5.91**	> 1.0 × 10^-6^

HAK-4	Z-Phe-Ala-BTh	**35.1 ± 6.75**	> 1.0 × 10^-5^

HAK-5	Z-Phe-Pro-Th	**35.2 ± 12.92**	**1.1 × 10^-10^**

HAK-6	Z-Lys-Pro-BTh	**38.8 ± 5.26**	**4.1 × 10^-9^**

HAK-7	Z-Asp-Pro-BTh	**52.8 ± 3.91**	**1.2 × 10^-8^**

HAK-8	Z-Arg-Pro-BTh	96.8 ± 10.69	**3.2 × 10^-9^**

HAK-9	Z-Phe-Phe-BTh	182.8 ± 25.41	**> 1.0 × 10^-5^**

IL-6 values are expressed as mean ± SD. IL-6 release of vehicle-treated cells was set to 100% (n = 2). Seven out of 9 HAK compounds suppressed significantly the OSM-induced IL-6 release. There was no intimate correlation between significant IL-6 reduction (**bold**) and the potency of HAK compounds to inhibit activity of recombinant prolyl endopeptidase (**bold**), presented as Ki values. All amino acids were used in L-configuration. Z, benzyloxycarbonyl, BTh, benzothiazole, Th, thiazole.

**Figure 3:**
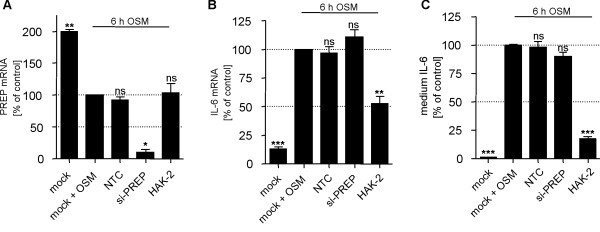
**Effect of PREP knock down on OSM-induced IL-6 expression in U343 cells**. PREP knock down does not suppress OSM-induced IL-6 expression in U343 cells, whereas the compound HAK-2 reduces IL-6 levels under identical test conditions. U343 cells were transfected with PREP-specific siRNA and non-target control siRNA (NTC) or vehicle (control). After 48 h cells were transfected again for further 24 h followed by OSM-stimulation for 6 h. As positive control, only vehicle transfected cells were treated with compound HAK-2 (20 μM). Expression levels of PREP (A) and IL-6 mRNA (B) were measured by qRT-PCR, while conditioned media were used for quantification of IL-6 release by ELISA (C). ns, not significant; **p < 0.01; ***p < 0.001.

While PREP inhibitor compounds HAK-1, HAK-2, HAK-5, HAK-6 and HAK-7 significantly reduced OSM-induced IL-6 secretion, there was no intimate correlation between the extent of PREP inhibition and the potency to suppress the IL-6 expression for different HAK compounds (Table 1). For example, compound HAK-8 is a potent PREP inhibitor but does not reduce OSM-stimulated IL-6 secretion (Table 1). On the other hand, compounds HAK-3 and HAK-4 are poor PREP inhibitors but significantly reduced OSM-stimulated IL-6 secretion (Table 1). This indicates that HAKs reduce IL-6 secretion independent from their PREP-inhibiting activity. In contrast to PREP inhibition, the proline residue at position P_1 _can be replaced by other amino acid residues like alanine or leucine without loss the bioactivity to reduce IL-6 expression. To clarify the role of PREP in the regulation of IL-6 expression, PREP was knocked down by siRNA technique in U343 cells. The remaining mRNA expression level of PREP was lower than 15% in comparison to mock and to non-target control (NTC) siRNA sample (Figure 3A). Interestingly, 6 h after onset of OSM stimulation, a 2-fold higher PREP mRNA level was obtained in non OSM-treated cells compared to OSM-stimulated NTC and mock samples. The biological basis of PREP up-regulation under these experimental conditions is not known, but could involve similar mechanisms contributing to induction of PREP in glial cells in experimental animals [39]. Compound HAK-2 did not have an effect on this phenomenon, allowing to investigate the effect of siRNA-mediated PREP knock down on OSM-stimulated IL-6 expression. Contrary to compound HAK-2, neither IL-6 mRNA level nor IL-6 protein level in the conditioned medium was significantly reduced after specific knock down of PREP (Figures 3B and 3C). This result strongly indicates that PREP is not involved in regulation of IL-6 expression by HAKs. Therefore, we conclude that HAKs exert their effects on IL-6 expression independent from PREP inhibition by modulating at least a second molecular target.

### Effect of HAK compounds on OSM-induced IL-6 mRNA expression

**Figure 4:**
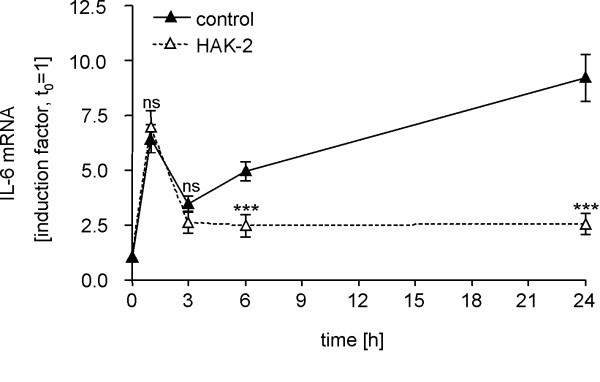
**Time course of OSM-induced IL-6 mRNA expression in U343 cells treated with compound HAK-2**. Compound HAK-2 completely suppresses exclusively the second IL-6 mRNA expression peak. U343 cells were stimulated with 100 ng/ml OSM and treated with 0.1% DMSO as solvent control (solid line, black triangle) or 20 μM HAK-2 (dashed line, white triangle). Samples were harvested at time points indicated to extract total RNA. IL-6 mRNA expression was analyzed by qRT-PCR. All data points are presented as mean ± SD, and were obtained from experiments carried out in quadruplicate. Statistical analysis were performed between control and HAK-2 samples from same time point. ns, not significant; ***p < 0.001.

To reveal whether bioactivity of HAK compounds is based on suppression of IL-6 protein biosynthesis or on interference with IL-6 mRNA expression OSM-treated U343 cells were incubated with 20 μM of compound HAK-2 for different periods of time. Time course analyses revealed a strong inhibition of the OSM-induced IL-6 mRNA expression by compound HAK-2 (Figure 4). Notably, only the second peak in IL-6 mRNA synthesis at 6 h post stimulation was affected, whereas the first peak 1 h post stimulation was insensitive to HAK-2 treatment. Additional experiments demonstrated suppression of OSM-induced IL-6 expression in U343 cells by HAK compounds even after delayed onset of treatment 6 h after OSM stimulation (data not shown). Therefore, it is very likely that the relevant molecular target of HAK compounds is involved in the OSM-induced signal transduction process not earlier than 6 h after onset of the stimulation.

Furthermore, IL-6 mRNA decay experiments were performed with actinomycin D, a transcription arresting agent, to study whether the strong inhibition of IL-6 mRNA expression by HAK compounds was based on modified mRNA stability. No difference in mRNA stability was observed between treated and non-treated cells (data not shown), demonstrating that the HAK compound-mediated suppression of IL-6 mRNA is most likely due to inhibition of transcription rather than modified mRNA stability.

### Suppression of LPS-induced IL-6 release by HAK compounds in primary murine astrocytes

**Figure 5:**
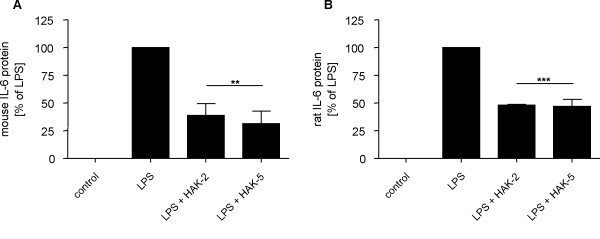
**Suppression of LPS-induced IL-6 release in primary murine astrocytes by selected HAK compounds**. Compounds HAK-2 and HAK-5 significantly reduce LPS-induced IL-6 release in primary mouse (A) and rat astrocytes (B). Astrocytes derived from newborn mice and rats were cultivated for 33 days followed by stimulation with 1 μg/ml LPS and treatment with compounds HAK-2 and HAK-5 (20 μM). After 24 hours conditioned media were analyzed with IL-6 specific ELISA. Data (mean ± SD) were obtained from experiments carried out in quadruplicate and statistical analysis was performed using the paired t test. Amount of IL-6 in LPS-treated control sample was set to 100%. ns, not significant; **p < 0.01; ***p < 0.001.

To analyze whether inhibition of OSM-induced IL-6 expression is a cell line-specific effect or a common feature of HAK compounds and valid in general, primary murine astrocytes were treated with HAK compounds. In contrast to human U343 glioma cells, OSM treatment did not lead to an increased IL-6 expression in mouse and rat primary astrocytes (data not shown). However, LPS significantly induced IL-6 release into the conditioned medium of mouse and rat astrocyte cultures (Figure 5).

Primary murine astrocytes stimulated with 1 μg/ml LPS were co-treated with compounds HAK-2 and HAK-5 at a concentration of 20 μM each for 24 h. The observed effect of compounds HAK-2 and HAK-5 on LPS stimulation was similar to that of OSM-induced IL-6 expression in human U343 glioma cells (Figures 5A and 5B). In comparison to untreated samples LPS-induced IL-6 expression was reduced by 60% in mouse and 50% in rat astrocytes by both HAK compounds. Thus, suppression of both, LPS- and OSM-induced IL-6 expression in different cell types by structurally related compounds is another indication for strong potency of HAK compounds to target neuroinflammatory processes.

### Potent inhibition of IL-6 upregulation by compound HAK-2 *in vivo*

Based on the results obtained from primary murine astrocytes, we reasoned that HAK compounds might also suppress elevated IL-6 expression *in vivo*. Therefore, bioactivity of the compound HAK-2 was analyzed in the LPS-induced mouse septic shock model. In preparation of this *in vivo *study, selected HAK compounds were characterized in detail concerning brain bioavailability. It was demonstrated by quantitative analysis of mouse plasma and brain samples using LC-MS/MS that the compounds are bioavailable and able to pass the blood brain barrier (data not shown). For further *in vivo *investigations compound HAK-2 with a logBB of -0.22 was selected.

**Figure 6:**
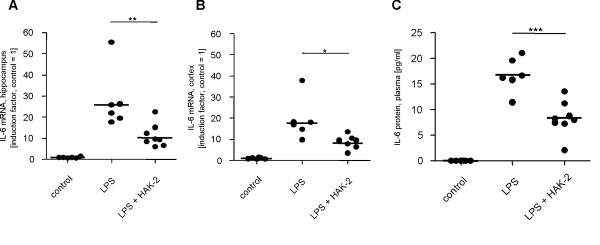
**Suppression of LPS-induced IL-6 expression *in vivo *by compound HAK-2**. C57/B6 mice (5 months old) were injected intraperitoneally (i.p.) with saline (control group, n = 6) or with 1 mg LPS/kg body weight (LPS group, n = 6). Compound HAK-2 (10 mg/kg body weight) was co-injected i.p. with LPS (LPS + HAK-2 group, n = 8). After 2 hours treatment, samples from hippocampus (A), cortex (B) and plasma (C) were prepared and analyzed by qRT-PCR and ELISA, respectively. Data obtained were normalized by means of mRNA level of 3 reference genes (glyceraldehyde 3-phosphate dehydrogenase, glucose-6-phosphate dehydrogenase and hypoxanthine-guanine phosphoribosyltransferase). Results are shown as induction factors compared to those of saline-treated mice (given as factor 1). Statistically significant differences between LPS group and LPS + HAK-2 group were calculated using the unpaired t-test (*p < 0.05; **p < 0.01; ***p < 0.001).

Intraperitoneal injection of 1 mg/kg LPS into C57/B6 mice resulted in an acute elevation of IL-6 concentration in plasma, hippocampus and cortex 2 h post administration (Figures 6A-C). The plasma level of IL-6 protein was significantly reduced by 55% in mice treated with 5 mg/kg HAK-2 in parallel as compared to LPS alone (Figure 6C). Similar effects of HAK-2 treatment were observed for induced IL-6 mRNA in hippocampus (60%) and cortex (53%) (Figures 6A and 6B). These data clearly show the strong potency of HAK compounds to modify IL-6 expression *in vivo*.

### Effect of HAK compounds on OSM-mediated phosphorylation of signal transducer and activator of transcription 3 (STAT3) and extracellular signal-regulated kinase 1 (Erk1)

**Figure 7:**
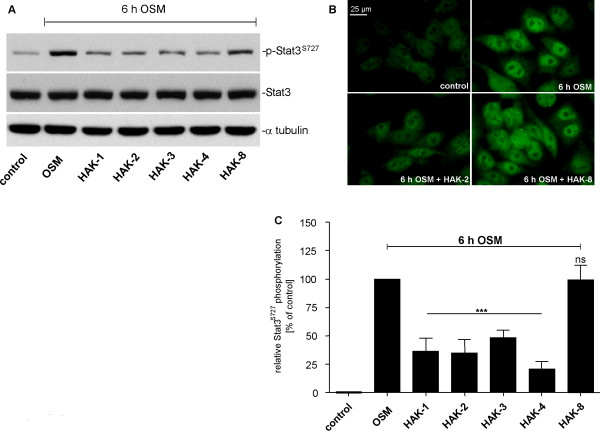
**Effect of selected HAK compounds on OSM-induced STAT3 phosphorylation at serine 727 in U343 cells**. Treatment of U343 cells with HAK compounds led to a significant reduction of OSM-induced pSTAT3^S727^, while STAT3 expression was not affected. For western blot analysis (A) 0.25 × 10^6 ^cells co-treated for 6 h with OSM (100 ng/ml) and selected HAK compounds (20 μM) were extracted and analyzed as described in experimental procedures. Blots (30 μg protein/lane) were probed with STAT3 and pSTAT3^S727^-specific antibodies (1:500, overnight). For immunocytochemistry (B) 0.1 × 10^6 ^cells were grown on cover slips and treated identically as described above. Post treatment, cells were fixed in methanol and labeled with a pSTAT3^S727^-specific antibody (1:200, overnight). From each sample 15 images were taken by means of fluorescence microscope and app. 250 cells/sample were densitometrically analyzed by MetaMorph imaging software (C). Mean signal intensity of OSM-treated control cells was set to 100%. All data are presented as mean ± SD from experiments carried out in triplicate. ns, non significant; ***p < 0.001.

The activation of the OSM signaling cascade resulting in the stimulation of IL-6 expression is known to involve intracellular phosphorylation events [31,40,41]. Therefore, effects of HAK compounds on the OSM-induced phosphorylation of signal transducer and activator of transcription 3 (STAT3) and extracellular signal-regulated kinase 1 (Erk1) were investigated. Since HAK compounds were shown to be bioactive 3 to 6 h post stimulation, U343 cells were incubated with OSM for 6 h. In contrast to non-stimulated control, OSM induced phosphorylation of Erk1 as well as STAT3 6 h post stimulation. Interestingly, HAK compounds suppressed STAT3 phosphorylation at serine 727 (pSTAT3^S727^) (Figure 7), but neither phosphorylation of pSTAT3^Y705 ^nor pErk1/2^T202/Y204 ^(data not shown). Compound HAK-8 showed a significantly lower effect on pSTAT3^S727 ^phosphorylation. This observation correlates well with the missing effect of compound HAK-8 on IL-6 expression as shown in Table 1. HAK compound-specific suppression of OSM-induced pSTAT3^S727 ^was confirmed by immunocytochemistry (Figures 7B-C). U343 cells cultivated on cover slips were treated identically as described before. In figure 7A an example of HAK compound efficiency to suppress nuclear pSTAT3^S727 ^phosphorylation 6 h post OSM stimulation is shown. Nuclear localization was confirmed by DAPI staining (data not shown). In figure 7C densitometric results of at least 3 independent experiments are summarized. While compounds HAK1-7 significantly suppressed the OSM-mediated phosphorylation of pSTAT3^S727^, compound HAK-8 did not. Results from western blot analyses and immunocytochemistry are strongly correlating with each other. Thus, it appears that the IL-6 reducing bioactivity of HAK compounds is most likely based on suppression of STAT3 phosphorylation at serine 727.

### STAT3 and NF-κB subunit p65 are forming a OSM-dependent complex which is sensitive to HAK compounds

**Figure 8:**
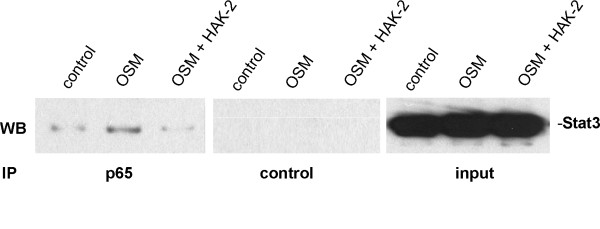
**Effect of compound HAK-2 on OSM-induced STAT3/p65 complex formation in U343 cells**. OSM treatment led to increased STAT3/p65 complexation, which is suppressed by compound HAK-2. U343 cells were stimulated with 100 ng/ml OSM and co-treated with compound HAK-2 (20 μM) for 6 h. After treatment, cells were lysed and whole-cell extracts were immunoprecipitated with a polyclonal antibody against the NF-κB subunit p65 or vehicle (control). Immunoblots were prepared and probed with polyclonal antibody against STAT3. Data from a representative experiment (n = 2) are shown.

Normally, STAT3 is activated by phosphorylation at tyrosine 705, which induces dimerization, nuclear translocation and DNA binding. In contrast to other transcription factors like NF-κB, Creb and c/EBPb, STAT3 can not bind directly to the IL-6 promoter, because there are no STAT3 binding elements present. It is known from literature that several forms of interactions and cross talks between NF-κB and STAT3 exist. For instance, recent studies have shown a physical interaction between STAT3 and NF-κB [42,43]. The importance of pSTAT3^S727 ^for protein interactions are under discussion [42,44]. However, there is no information so far on the role of pSTAT3^S727 ^for the interaction with NF-κB. To characterize a possible OSM-induced and p^S727^-dependent complex formation between STAT3 and NF-κB, we performed co-immunoprecipitation of p65 followed by western blotting for STAT3. In this experiment, U343 glioma cells were stimulated with OSM for 6 h in the absence or presence of compound HAK-2. The immunoprecipitation analysis showed that STAT3 and p65 were co-immunoprecipitated in a OSM-dependent manner (Figure 8). Furthermore, treatment of human U343 glioma cells with compound HAK-2 led to a clear reduction of p65-mediated STAT3 co-immunoprecipitation. Moreover, these data confirm OSM- and phosphorylation-dependent complex formation between STAT3 and p65, which is sensitive to HAK compounds.

## Discussion

Neuropathological situations with extended astroglial activation are associated with increased levels of pro-inflammatory mediators including IL-6. *In vitro *and *in vivo *studies have demonstrated that IL-6 plays a pivotal role in the initiation of neuroinflammatory cascades and in secondary neuronal cell death [6,45]. Thus, prevention of the neuroinflammatory response to primary lesions has a neuroprotective potential.

The present study was performed to identify new small molecular weight inhibitors acting on the pathway that results in IL-6 expression. For screening of our in-house compound libraries the human glioblastoma cell line U343 was used, because glioblastoma cell lines were shown to respond with increased IL-6 expression to different neuroinflammatory stimuli like LPS [27], Substance P [46], tumor necrosis factor α, interleukin-1β, leukemia-inhibitory factor and OSM [30]. Analysis of conditioned media revealed, that in our experimental setup only OSM treatment significantly induced the expression of IL-6 in human U343 glioma cells. This result is consistent with published data, showing that U343 cells express the OSM-receptor components LIFR and OSMRβ as well as the common signal transducer gp130 [47]. Furthermore, the OSM-mediated activation of signal components of the Jak/STAT- and MAPK-pathways was described for U343 and U373 glioma cells, respectively [32]. We observed a biphasic induction pattern of OSM-induced IL-6 mRNA expression, which was described earlier also for human U373 astroglioma cells [32]. The time-course is characterized by a first strong, rapid and transient IL-6 mRNA expression peak at 1 h followed by a second one at 6 h with a less strong, but prolonged induction. The same type of expression pattern was observed for tissue factor mRNA in OSM-treated smooth muscle cells [48]. Thus, biphasic induction seems to be an OSM-specific feature with general relevance for OSM action.

All potent inhibitors of IL-6 secretion identified in the compound library screen (see Table 1) belong to the chemical class of HAK and are structurally related to inhibitors of PREP [38]. This observation is in line with the hypothesis, that PREP is involved in regulation of intracellular protein transport and secretion [49]. However, there was no correlation between PREP siRNA- (knock-down) and pharmacological inhibition of PREP on one hand and the potency of these compounds to suppress the OSM-induced IL-6 expression on the other. Furthermore, our data on the temporal profile of IL-6 suppression suggest that the bioactivity of HAK compounds is most likely based on interference with IL-6 mRNA synthesis but not on disturbed intracellular transport and secretion mechanisms. Therefore, PREP can be excluded as the IL-6 relevant molecular target of HAKs and HAK compounds appear to interact with at least one further molecular target. Interestingly, only the second IL-6 mRNA peak was affected by HAKs indicating that the molecular target of HAK compounds is involved 3 to 6 h post OSM stimulation at earliest. Theoretically, the biological target of HAKs can pre-exist in untreated cells or be induced by OSM treatment and subsequently incorporated in signaling pathways. Notably, in an experiment analyzing the puromycin sensitivity of OSM-induced IL-6 mRNA expression, it was demonstrated that OSM induces the protein synthesis of signaling molecules essential for the second IL-6 mRNA expression peak [32]. Whereas puromycin completely abrogated the second IL-6 expression peak it showed no effect on the first OSM-induced IL-6 mRNA peak. This demonstrates a requirement for de novo protein synthesis exclusively for the second IL-6 expression peak of this biphasic response signaling. The relationship between HAK-mediated suppression of OSM-induced IL-6 release and the effect of HAK compounds exclusively on the second mRNA peak suggests that more than 75% of secreted IL-6 is based on the second phase of OSM-induced IL-6 mRNA expression. Thus, the mRNA induced in the first phase appears to have regulatory functions rather than acting as a template in protein synthesis. Such a regulatory role of mRNA molecules was recently described by Poliseno et. al. [50] showing that mRNA molecules from pseudogenes or long non-coding RNAs can act as competitive endogenous RNAs sequestering microRNA molecules.

To elucidate whether the HAK-mediated suppression of OSM-induced IL-6 expression is cell line-specific or valid in general, experiments with primary murine astrocytes were performed. In contrast to human U343 glioblastoma OSM did not induce IL-6 expression in mouse and rat primary astrocytes. However, LPS, known to act as a powerful stimuli of cytokines [51], significantly increased IL-6 expression in primary murine astrocytes. Co-treatment with HAK compounds markedly suppressed levels of OSM-stimulated IL-6 expression in both rat and mouse astrocytes. These data demonstrate that the anti-inflammatory bioactivity of HAKs is not limited to a single OSM-based cell culture model but also valid for a series of pathophysiological conditions contributing to neuroinflammation and neurodegeneration.

We were also interested to reveal whether HAK compounds are bioactive under inflammatory conditions *in vivo*. For this study, compound HAK-2 was selected based on its beneficial features concerning toxicity, bioavailability and blood brain barrier passage. In accordance with the data obtained from primary murine astrocytes, compound HAK-2 significantly suppressed LPS-induced IL-6 levels in brain- and plasma-derived samples from septic mice. This result strongly indicates the anti-inflammatory potency of HAK compounds *in vivo *for possible treatment of central nervous system diseases.

To get more information about the underlying molecular mechanism of HAK bioactivity, the signal-transduction pathways involved in OSM-mediated IL-6 expression were dissected in more detail. Interestingly, LPS- and OSM-induced signal pathways are based on the same molecular mechanism such as STAT3 or NF-κB activation [31,52], indicating that HAK compounds may target a common cellular event. Two major signaling cascades the JAK/STAT as well as the MAPK pathways are switched on by binding of OSM to the receptor heterodimers OSMR/gp130 or LIFR/gp130 [53]. Subsequent activation of signal tyrosine kinases of the JAK family leads to phosphorylation of pivotal signal molecules such as STAT3 and Erk1 and 2 respectively [31,32]. The essential role of receptor subunits as well as of downstream signaling molecules as STAT3, Erk1 and p65 for OSM-triggered IL-6 expression in U343 cells was confirmed by siRNA-based knock-down experiments (data not shown). Furthermore, Erk1/2 and STAT3 were phosphorylated 6 h post OSM treatment, which was identified as the critical time point for the HAK bioactivity. Immunoblotting and immunofluorescence experiments revealed that neither OSM-induced pErk1/2^T202/Y204 ^phosphorylation nor pSTAT3^Y705 ^phosphorylation were modified by HAK compounds. However, HAK treatment led to a significant reduction of OSM-stimulated pSTAT3^S727 ^phosphorylation. Importantly, the HAK-based inhibition profiles for IL-6 expression and pSTAT3^S727 ^phosphorylation are strongly correlating with each other. Thus, suppression of OSM-induced phosphorylation of pSTAT3^S727 ^is most likely the relevant molecular mechanism of the HAK compound bioactivity to suppress IL-6 expression. In contrast to pSTAT3^Y705^, which is essential for dimerization, nuclear translocation and DNA binding [54,55], the physiological role of pSTAT3^S727 ^is discussed controversially [56-59]. Depending on the specific promoter and/or the cellular context pSTAT3^S727 ^can influence transcriptional activity of target genes [60,61].

However, in the case of the IL-6 promoter, where activated NF-κB binds directly to DNA, no cis-regulatory elements for STAT3 binding were identified so far [62-64]. Based on these observations, we hypothesize that pSTAT3^S727 ^may regulate IL-6 gene expression by an alternative pathway. It is known that STAT3 is complexed with transcription factors such as c-Jun, c-Fos, forkhead and endothelial cell-derived zinc finger protein, respectively [65-68]. Furthermore, it was shown that physical interaction of the STAT3 DNA-binding domain with the NF-κB subunit p65 led to a reduced promoter activity of inducible nitric oxide synthase gene [69].

Together, these findings strongly suggest that physical interaction between STAT3 and p65 may result in a functional coupling important for the STAT3-dependent regulation of p65 responsive genes. Indeed, we here demonstrated by co-immunoprecipitation that p65 and STAT3 interact with each other in an OSM-dependent manner. Noteworthy, the OSM-stimulated STAT3 and p65 complex formation is quite sensitive against treatment with HAK compounds. This supports our hypothesis and indicates for the first time a regulatory function for pSTAT3^S727 ^in OSM-triggered STAT3/NF-κB interaction.

In summary, HAK compounds selectively target the expression of genes with promoters co-regulated by pSTAT3^S727^-dependent signaling. Based on this mechanism, kinases phosphorylating STAT3 at serine 727 such as MAPKs, mTOR, NLK and PKCδ may represent direct molecular targets of HAK compounds. Thus, further studies are required to identify the precise molecular mechanisms and the neuroinflammatory-related genes sensitive to HAK-treatment. This will enable the therapeutic development of HAK compounds for treatment of neurological diseases including Alzheimer's disease, multiple sclerosis, Parkinson's disease and traumatic brain injury.

## Conclusions

In the present study, we identified and characterized for the first time HAK compounds as potent inhibitors of OSM- and LPS-induced IL-6 expression *in vitro *and *in vivo*. The molecular trigger of HAK bioactivity is most-likely a selective suppression of STAT3 phosphorylation at serine 727. Pathological upregulation of astrocytic IL-6 expression is known to play a pivotal role in onset and progression of neurological diseases including Alzheimer's disease, multiple sclerosis, Parkinson's disease and traumatic brain injury. In conclusion, we propose that HAK compounds represent a new potent class of drugs for treatment or prevention of neuroinflammation and subsequent neurodegeneration.

## Abbreviations

**HAK: **heteroarylketone; **IL-6: **interleukin-6; **LPS: **lipopolysaccharide; **MAPK: **mitogen-activated protein kinase; **NF-κB: **nuclear factor kappa B; **OSM: **Oncostatin M; **PREP: **prolyl endopeptidase; **STAT3: **signal transducer and activator of transcription.

## Competing interests

IS, CE, AJN, KM, AK and AM are employees of Probiodrug AG. HUD serves as CSO of Probiodrug AG.

## Authors' contributions

IS initiated the project and wrote the paper. IS, AJN, CE, UZ, KM, AM and SR performed the experiments and analyzed the data. AK assisted with the design and interpretation of qRT-PCR experiments. AK, HUD and SR participated in study design and coordination as well as drafted and revised the manuscript. All authors read and approved the final manuscript.
